# T cells in multiple myeloma display features of exhaustion and senescence at the tumor site

**DOI:** 10.1186/s13045-016-0345-3

**Published:** 2016-11-03

**Authors:** Claudia Zelle-Rieser, Shanmugapriya Thangavadivel, Rainer Biedermann, Andrea Brunner, Patrizia Stoitzner, Ella Willenbacher, Richard Greil, Karin Jöhrer

**Affiliations:** 1 Tyrolean Cancer Research Institute, Innrain 66, 6020 Innsbruck, Austria; 2 Department of Orthopedic Surgery, Medical University of Innsbruck, Anichstraße 35, Innsbruck, Austria; 3 Department of Pathology, Medical University of Innsbruck, Müllerstraße 44, Innsbruck, Austria; 4 Department of Dermatology, Venereology and Allergology, Medical University of Innsbruck, Anichstraße 35, Innsbruck, Austria; 5 Department of Internal Medicine V, Medical University of Innsbruck, Anichstraße 35, Innsbruck, Austria; 6 Salzburg Cancer Research Institute (SCRI), Müllner Hauptstraße 48, 5020 Salzburg, Austria; 7 Third Medical Department at The Paracelsus Medical University Salzburg, Müllner Hauptstraße 48, Salzburg, Austria

**Keywords:** Multiple myeloma, Immune-checkpoint molecules, T cell exhaustion, T cell senescence, Bone marrow

## Abstract

**Background:**

Multiple myeloma is an incurable plasma cell malignancy that is mostly restricted to the bone marrow. Cancer-induced dysfunction of cytotoxic T cells at the tumor site may be responsible for immune evasion and therapeutical failure of immunotherapies. Therefore, enhanced knowledge about the actual status of T cells in myeloma bone marrow is urgently needed. Here, we assessed the expression of inhibitory molecules PD-1, CTLA-4, 2B4, CD160, senescence marker CD57, and CD28 on T cells of naive and treated myeloma patients in the bone marrow and peripheral blood and collected data on T cell subset distribution in both compartments. In addition, T cell function concerning proliferation and expression of T-bet, IL-2, IFNγ, and CD107a was investigated after in vitro stimulation by CD3/CD28. Finally, data was compared to healthy, age-matched donor T cells from both compartments.

**Methods:**

Multicolor flow cytometry was utilized for the analyses of surface molecules, intracellular staining of cytokines was also performed by flow cytometry, and proliferation was assessed by ^3^H-thymidine incorporation. Statistical analyses were performed utilizing unpaired *T* test and Mann-Whitney *U* test.

**Results:**

We observed enhanced T cell exhaustion and senescence especially at the tumor site. CD8+ T cells expressed several molecules associated with T cell exhaustion (PD-1, CTLA-4, 2B4, CD160) and T cell senescence (CD57, lack of CD28). This phenotype was associated with lower proliferative capacity and impaired function. Despite a high expression of the transcription factor T-bet, CD8+ T cells from the tumor site failed to produce IFNγ after CD3/CD28 in vitro restimulation and displayed a reduced ability to degranulate in response to T cell stimuli. Notably, the percentage of senescent CD57+CD28− CD8+ T cells was significantly lower in treated myeloma patients when compared to untreated patients.

**Conclusions:**

T cells from the bone marrow of myeloma patients were more severely impaired than peripheral T cells. While our data suggest that terminally differentiated cells are preferentially deleted by therapy, immune-checkpoint molecules were still present on T cells supporting the potential of checkpoint inhibitors to reactivate T cells in myeloma patients in combination therapies. However, additional avenues to restore anti-myeloma T cell responses are urgently needed.

**Electronic supplementary material:**

The online version of this article (doi:10.1186/s13045-016-0345-3) contains supplementary material, which is available to authorized users.

## Background

Treatment of cancer aims at the deletion of all malignant cells. Besides minimizing the bulk of tumor cells by conventional therapy, the cytotoxic ability of activated T cells is central to tumor eradication and cancer cure. T cell anergy, exhaustion, and/or senescence are characterized by elevated levels of multiple inhibitory molecules, impaired effector cytokine production, loss of proliferative capacity, impaired cytotoxicity, and altered use of key transcription factors including T-bet (reviewed in [1–3]). T cell exhaustion occurs after chronic antigenic exposure and prevents optimal control of tumors. Modulation of molecules and pathways overexpressed in the exhaustion phase could reverse this dysfunctional state and reinvigorate immune response [4, 5]. Senescent T cells are late differentiated memory/effector T cells which lack CD28 and gain CD57 and regulatory receptor expression. They rather express CD45RA than CD45RO, are in cell-cycle arrest, and have enhanced secretion of inflammatory cytokines [6]. Most studied inhibitory molecules which function as negative regulators of lymphocyte activation are PD-1 (CD279) and CTLA-4 (CD152). Natural killer cell receptor 2B4 (CD244), glycoprotein CD160, TIM3, Lag3, and many others have also been shown to contribute essentially to the regulation of T cell activity [2, 7]. Blocking immune checkpoint molecules achieved promising results in the treatment of solid cancers; however, targeting these molecules in hematologic malignancies is relatively understudied [8–10]. First promising results with an anti-PD-1 antibody in the treatment of refractory Hodgkin’s lymphoma were recently published [11–13], and studies are under way to investigate the effectiveness of combinations with low-dose glucocorticoids. Recently, data on immune checkpoints such as PD-1 expression of myeloma patients have been published. In addition, the potential of PD-1 blocking antibodies to reactivate diseased T cells were investigated in vitro and proved this molecule as promising candidate for immunotherapy [14–16]. In vivo, however, these PD-1 inhibitors appear to work only in combination with immunomodulatory drugs in myeloma patients, and first positive results have been presented at the ASH 2015 meeting by J. San Miguel and A.Z. Badros.

Multiple myeloma is a hematologic cancer characterized by the accumulation of malignant plasma cells within the bone marrow. Despite the advances in therapy by the introduction of novel immunomodulatory agents and proteasome inhibitors, this cancer remains largely incurable. The immunogenic nature of multiple myeloma is underlined by the observation of disease remission after allogeneic stem cell transplantation or donor lymphocyte infusion, possibly caused by graft-versus-myeloma effects [17, 18]. Delineating the tumor-driven defects of the immune system in myeloma in more depth will contribute to the development of novel immuno-therapeutical strategies.

Defects in T cell distribution and function have been consistently documented in this disease, including a reduction of peripheral blood CD4 and CD8 T cells, inversion of the CD4/CD8 ratio, abnormal Th1/Th2 ratio, downregulation of signal transduction components, and abnormal T cell responses [19–22]. Intriguingly, the majority of studies so far focused on immunological alterations in the peripheral blood of myeloma patients despite the fact that myeloma cells almost exclusively grow and spread within the bone marrow. So far, little is known regarding the composition and activity of the immune system in myeloma bone marrow. In this study, we investigated the expression of inhibitory molecules on effector T cells as well as their function in response to in vitro stimulation by antibodies against CD3/CD28. We studied both T cells from peripheral blood and from the bone marrow of myeloma patients and healthy, age-matched donors in order to elucidate systemic and local tumor-mediated alterations.

We demonstrate that, especially at the tumor site, both T cell exhaustion and T cell senescence might play an important role in supporting tumor growth. Moreover, we show data on the T cell composition in refractory patients and how therapy might change T cell suppressive molecules.

## Methods

### Sample collection

Blood samples and bone marrow aspirates from the posterior iliac crest from the same patient were obtained from 16 newly diagnosed patients (MM naive) and 6 patients treated with immunomodulatory drugs and dexamethasone (MM Tx) at the Department of Internal Medicine, University Hospital of Innsbruck, Austria. Peripheral blood samples and bone marrow aspirates from the proximal femur of the same individuals were taken from age-matched healthy donors in the course of hip arthroplasty at the Department of Orthopedic Surgery, University Hospital of Innsbruck (*n* = 12). According to a recent publication, T cell subset distribution is comparable between samples from the posterior iliac crest and proximal femur [23]. Healthy subjects were screened for the presence of viral infections and any bone marrow abnormalities and did not receive immunomodulatory drugs or suffer from diseases known to influence the immune system, including autoimmune diseases or cancer.

### Cell isolation from human PB and BM aspirates

Peripheral blood mononuclear cells (PBMC) and bone marrow mononuclear cells (BMMC) were separated by density gradient centrifugation (GE Healthcare Life Sciences) and either used immediately (for flow cytometric analyses) or cryopreserved for later functional analysis. Recovery rates from frozen T cells were above 85 %.

### Immunofluorescence staining and flow cytometric analysis

Staining of surface antigens was performed in erythrocyte-free samples (blood samples and bone marrow aspirates) by multicolour staining using fluorescently labeled CD3 (clone UCHT1), CD4 (RPA-T4), CD8 (RPA-T8), CD28 (CD28.2), CD45 (2D1), CD45RA (HI100), CD57 (NK-1), CD62L (DREG-56), CD152/CTLA-4 (BNI3), CD160 (BY55), CD244/2B4 (2-69), CD279/PD1 (MI-H4) (all from BD, Heidelberg, Germany). For staining of intracellular proteins, PBMC and BMMC were stimulated with anti-CD3 (2 μg/ml) and anti-CD28 (5 μg/ml) (both from eBioscience/Affymetrix) for 6 or 24 h. Ten micrograms per milliliter Brefeldin A (Sigma-Aldrich) were added to the cell cultures for the last 4 h of incubation. Intracellular staining was carried out using CytoFix/CytoPerm kit, according to manufacturer’s instructions (BD). All antibodies against cytokines (IL-2, clone 5344.111; IFNγ, B27), T-bet (O4-46), and CD107a (H4A3) were purchased from BD. Flow cytometry was performed on a BD FACS Canto II flow cytometer with subsequent analysis using FACS DIVA Software 7.0. The analysis was performed after gating on single viable cells after 7-AAD staining.

### T cell proliferation and degranulation

Cell proliferation was measured by culturing BMMC at a density of 2 × 10^6^/ml in RPMI1640/10 % FCS together with anti-CD3 (2 μg/ml) and anti-CD28 (5 μg/ml) antibodies. ^3^H-thymidine (Hartmann Analytic) was added for the last 16 h of stimulation, cells were harvested, and ^3^H-thymidine incorporation was measured on a ß-counter (Beckman Coulter, USA). The proliferation index was calculated as ratio of stimulated T cells and unstimulated controls. For assessment of degranulation, cells were stimulated with anti-CD3 and anti-CD28 as above with the addition of 10 μg/ml anti-CD107a antibody for 6 h at 37 °C. For assessment of CD8+ T cell activation/proliferation, 5 × 10^6^ cells/ml (BMMC) were washed and stained with 0.4 μM carboxyfluorescein succinimidyl ester (CFSE; eBioscience/Affymetrix) for 5 min at room temperature in the dark. After quenching with FCS and washing with RPMI1640 and once with PBS, the cells were diluted at 2 × 10^6^/ml in RPMI1640/10 % FCS and stimulated with anti-CD3 and anti-CD28 antibodies for 72 h as described above.

### Statistical analysis

For all data sets which could be accurately modeled by a Gaussian distribution an unpaired *t* test was used for analysis of differences between groups; otherwise, the Mann-Whitney *U* test was used. *P* values of less than 0.05 were considered statistically significant (significance levels *p < 0.05, **p < 0.01, ***p < 0.001, n.s. not significant).

## Results

### Increased expression of PD-1, CTLA-4, CD160, and 2B4 on CD8+ T cells from the bone marrow of myeloma patients

We assessed expression of inhibitory molecules on T cells from the bone marrow (BM) and peripheral blood (PB) of myeloma patients and healthy, age-matched individuals by multicolour flow cytometry. Patients’ characteristics are summarized in Table 1.

**Table 1:** Donor characteristics

		Chemo-naive patients	Treated patients	Healthy controls
Number	Total	16	6	12
Age	Median	70	65	59
	Range	67–76	62–74	55–70
Sex	Male	8	3	7
	Female	8	3	5
International staging system	I	5	1	
	II	6	1	
	III	5	2	
	Unknown	0	2	
Durie-Salmon staging system	I	0	2	
	IIA	2	1	
	IIIA	10	2	
	IIIB	3	0	
	Unknown	1	1	
Immunoglobulin subtypes	IgG	8	4	
	IgA	5	1	
	Light chains	2	0	
	Unknown	1	1	
Immunoglobulin light chains	κ	11	4	
	λ	4	1	
	Unknown	1	1	

The patients were classified according to the International Myeloma Working Group (IMWG) criteria

In brief, both, CD3+CD4+ as well as CD3+CD8+ T cells exhibited enhanced expression of PD-1 in PB as well as BM of myeloma patients. This is in accordance with recently published data [15, 24]. We could also confirm enhanced expression of CTLA-4 [25], and we found it to be restricted to bone marrow T cells of myeloma patients. In general, the higher the numbers of inhibitory receptors which are present, the more severe T cells are exhausted. Therefore, we analyzed the expression of two additional inhibitory molecules which are suggested to play a role in T cell regulation, i.e., CD160 and 2B4. CD160 competes with BTLA for binding to herpes virus entry mediator (HVEM) and has been shown to negatively regulate TCR-mediated signaling [26]. Crosslinking of 2B4 on T cells decreases proliferation, and 2B4 expression is upregulated on exhausted T cells [27, 28]. In our analyses both, CD160 and 2B4, were significantly upregulated on bone marrow T cells of myeloma patients suggesting that the extent of local immune suppression might be even higher than estimated from the previous investigations. The percentages of T cells expressing the respective molecules are depicted in Table 2.

**Table 2:** Distribution of inhibitory molecules on CD4+ and CD8+ T cells in BM aspirates and PB of myeloma patients and healthy persons

	BM			PB		
	Healthy Median % (IQR)	Myeloma Median % (IQR)		Healthy Median % (IQR)	Myeloma Median % (IQR)	
CD4+ T cells						
PD-1+	44.4 (17.7–60.3)	64.5 (36.8–77.4)	*	47.2 (21.6–60.2)	66.6 (48.0–74.8)	*
2B4+	30.3 (7.3–43.2)	36.1 (28.4–47.3)	n.s.	33.7 (17.8–45.2)	33.4 (19.1–44.6)	n.s.
CD160+	29.4 (8.3–33.2)	30.8 (23.4–39.0)	n.s.	33.1 (10.5–37.3)	30.6 (16.8–38.9)	n.s.
CTLA-4+	43.5 (29.4–56.6)	60.3 (52.2–64.4)	*	48.8 (37.9–59.6)	56.7 (35.6–65.4)	n.s.
CD8+ T cells						
PD-1+	60.7 (50.8–69.4)	73.0 (65.7–86.8)	*	50.20 (42.0–66.7)	74.1 (57.8–81.9)	*
2B4+	42.0 (24.1–46.1)	58.5 (43.1–69.0)	*	56.50 (36.7–69.6)	59.4 (49.3–70.3)	n.s.
CD160+	38.7 (22.9–49.8)	56.4 (44.4–64.8)	*	44.30 (16.6–57.8)	49.4 (40.5–59.2)	n.s.
CTLA-4+	53.1 (44.9–59.6)	65.8 (61.9–67.9)	**	58.55 (43.5–64.2)	64.7 (60.6–71.8)	n.s.

Flow cytometric analyses of inhibitory molecules are presented as median percentage of cells expressing the respective molecules with IQR (*n* = 7–12). Statistical significance was calculated using Mann-Whitney *U* test

*n.s.* not significant

*p < 0.05; **p < 0.01

### T cell subsets are altered in myeloma bone marrow

PD-1 expression on T cells has recently been described as novel therapeutical target in myeloma disease [15]. In general, high levels of PD-1 expression are found on effector memory T cells [29]. In depth analyses of T cell subset distributions in myeloma are largely missing so far which prompted us to further analyze patients’ T cells according to CD45RA and CD62L levels. Concomitantly, we analyzed PD-1 levels on the different T cell subsets.

**Fig. 1:**
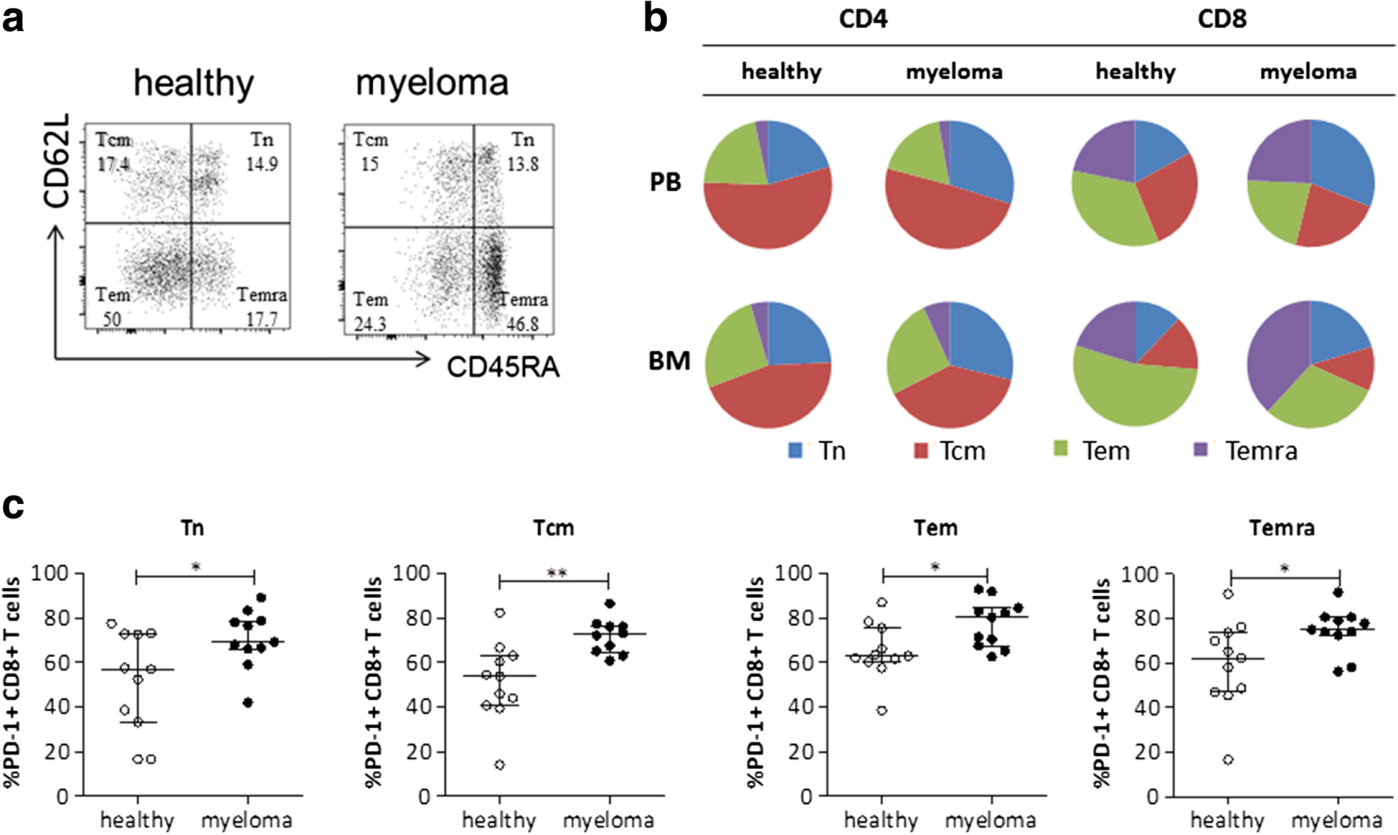
Accumulation of an activated CD8+ T cell population in the BM of myeloma patients. **a** The *dot plots* show the gating strategy as well as the proportion of human CD8+ T cell subsets of a representative healthy and myeloma BM sample. The *numbers* indicate the percentage of naive (Tn: CD45RA+CD62L+), central-memory (Tcm: CD45RA-CD62L+), effector memory (Tem: CD45RA−CD62L−) and effector (Temra: CD45RA+CD62L−) CD8+ T cells. **b** Pie charts summarize the median proportion of Tn, Tcm, Tem, and Temra in PB and BM for 12 healthy and 16 myeloma samples. **c** Percentages of PD-1 expressing CD8+ T cell subsets (Tn, Tcm, Tem, and Temra) of healthy, age-matched controls and myeloma patients were assessed by flow cytometry (*n* = 11; median with interquartile range (IQR) is shown). Significance levels *p < 0.05; **p < 0.01

Whereas percentage of CD4+ subsets did not vary significantly between healthy and diseased peripheral blood and bone marrow compartments, CD8+ subset distribution was strikingly altered. Briefly, we found a significant accumulation of effector T cells (Temra; CD45RA+CD62L−) and an even more pronounced decrease of effector memory T cells (Tem; CD45RA−CD62L−) within the CD8+ T cell pool in the myeloma bone marrow (Table 3, Fig. 1a, b). A trend for the increase of Temra cells as a consequence of aging has been described [30], and we found it to be further enhanced in myeloma patients. In addition, we found that PD-1 expression was significantly increased in all T cell subsets (Fig. 1c).

**Table 3:** T cell subset distribution in BM aspirates and PB of myeloma patients and healthy persons

	BM			PB		
	Healthy Median % (IQR)	Myeloma Median % (IQR)		Healthy Median % (IQR)	Myeloma Median % (IQR)	
CD4+ T cells						
CD45RA+/CD62L+ (Tn)	24.1 (13.2–39.10)	26.0 (14.5–36.4)	n.s.	18.1 (14.0–39.8)	25.7 (14.4–42.9)	n.s.
CD45RA−/CD62L+ (Tcm)	44.6 (37.2–48.2)	35.0 (19.4–52.6)	n.s.	48.8 (43.3–61.3)	42.4 (31.6–60.0)	n.s.
CD45RA−/CD62L− (Tem)	26.0 (22.1–32.2)	23.2 (9.2–36.3)	n.s.	18.7 (13.3–21.8)	15.6 (9.8–25.9)	n.s.
CD45RA+/CD62L− (Temra)	4.5 (2.4–6.2)	6.2 (2.0–16.8)	n.s.	2.9 (0.9–3.9)	2.4 (0.4–7.0)	n.s.
CD8+ T cells						
CD45RA+/CD62L+ (Tn)	11.3 (7.6–18.4)	18.5 (10.3–24.7)	n.s.	14.8 (9.2–28.2)	26.3 (15.7–53.5)	n.s.
CD45RA−/CD62L+ (Tcm)	13.2 (9.8–17.1)	10.2 (7.0–15.3)	n.s.	23.5 (18.6–34.5)	19.4 (12.5–36.9)	n.s.
CD45RA−/CD62L− (Tem)	49.6 (40.0–66.2)	27.2 (19.8–38.9)	**	30.1 (27.1–44.0)	18.5 (11.3–32.4)	*
CD45RA+/CD62L− (Temra)	18.7 (15.1–27.9)	34.5 (25.7–52.4)	**	19.0 (11.4–32.3)	20.6 (7.7–31.3)	n.s.

Percentages of the various T cell subsets in PB and BM were determined by flow cytometric analyses and are presented as median percentage with IQR (*n* = 12–16). Statistical significance was calculated using Mann-Whitney *U* test

*Tn* naïve T cell, *Tcm* central memory T cell, *Tem* effector memory T cell, *Temra* effector T cell, *n.s.* not significant

*p < 0.05; **p < 0.01

### Bone marrow CD8+ T cells of myeloma patients show functional defects

**Fig. 2:**
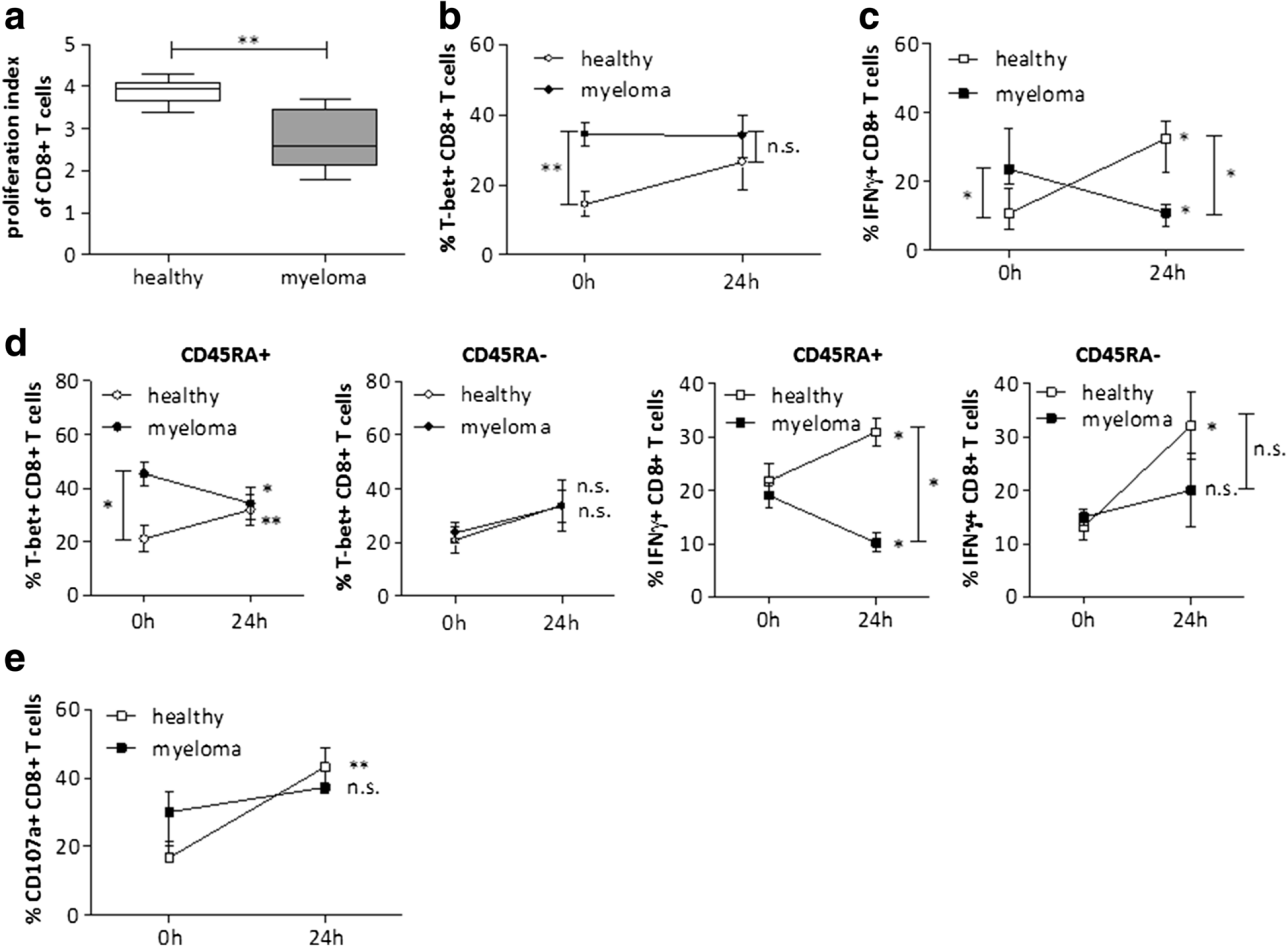
CD8+ T cells of myeloma patients show functional defects in proliferation and cytotoxicity. **a** Proliferation was assessed by ^3^H-thymidine incorporation after stimulating the cells for 72 h with anti-CD3/anti-CD28 antibodies. The proliferative index (^3^H-thymidine incorporation of stimulated T cells divided by ^3^H incorporation of unstimulated cells) is shown for BM CD8+ T cells from myeloma patients (*n* = 12) and from healthy controls (*n* = 6). **b** T-bet and **c** IFNγ expression was assessed by intracytoplasmic staining of total CD8+ BMMCs before (0 h) and after stimulation (24 h) of the cells (*n* = 7; median with IQR is shown). **d** T-bet and IFNγ expression on different T cell subtypes (CD8+ CD45RA+ or CD45RA−) was assessed by intracytoplasmic staining before (0 h) and after (24 h) stimulation with CD3/CD28 antibodies (*n* = 7; mean ± SEM is shown). **e** Surface levels of CD107a were detected by flow cytometry before (0 h) and after stimulation (24 h) (*n* = 3; median with IQR is shown). Significance levels: *p < 0.05; **p < 0.01; *n.s*. not significant

In the next set of experiments, we assessed the ability of BM CD8+ T cells to proliferate in response to T cell stimulation by anti-CD3 and anti-CD28 antibodies. Proliferation of CD8+ T cells from myeloma patients was significantly lower compared to healthy controls (Fig. 2a). To further investigate the involved mechanisms, we compared expression of various molecules in the above setting. First, we analyzed the transcription factor T-bet which plays a crucial role in T cell development, regulates effector function, and is essential for the induction of IFNγ [31]. Significantly more T-bet+ CD8+ T cells were detected in BM samples of myeloma patients. However, upon stimulation, healthy donor T cells upregulated T-bet whereas expression in myeloma T cells remained the same (Fig. 2b). The high constitutive expression of T-bet is consistent with a skewing towards CD8+ effector cell subsets as shown above. Physiologically, high T-bet expression goes along with enhanced cytokine production [32]. According to T-bet expression, the percentage of IFNγ-expressing cells was higher in unstimulated samples of myeloma patients compared to healthy donors. However, upon activation, expression was significantly downregulated in myeloma T cells whereas it was upregulated in healthy controls (Fig. 2c). This finding points to an activated but exhausted CD8+ T cell population in diseased BM. Interestingly, further in depth analyses of T-bet and IFNγ expression in T cell subsets revealed a significant decrease in the CD45RA+ CD8+ T cell compartment (Tn and Temra) of myeloma patients, whereas expression in CD45RA− CD8+ T cells (Tcm and Tem) was similar in healthy and myeloma people (Fig. 2d). Taking into account that effector T cells (Temra) represent about 60 % of the T cell CD45RA+ compartment in myeloma patients, we suggest that effector cell function is markedly impaired.

Cytotoxic T cell function also relies on their functional degranulation capacity. CD107a (LAMP1) has been described as a marker of cytotoxic CD8+ T cell degranulation and is strongly upregulated on the cell surface of this T cell type upon stimulation [33]. Interestingly, myeloma CD8+ T cells displayed high constitutive CD107a expression. Upon stimulation, healthy BM CD8+ T cells were able to mobilize intracellular CD107a to the membrane whereas degranulation of myeloma CD8+ T cells was impaired (Fig. 2e). These data also support a decreased cytotoxic activity of CD8+ T cells in multiple myeloma. Together, the impaired proliferative and cytotoxic capacity of T cells in BM of myeloma patients in response to activation suggests T cell exhaustion.

### Accumulation of a senescent T cell population in myeloma bone marrow

**Fig. 3:**
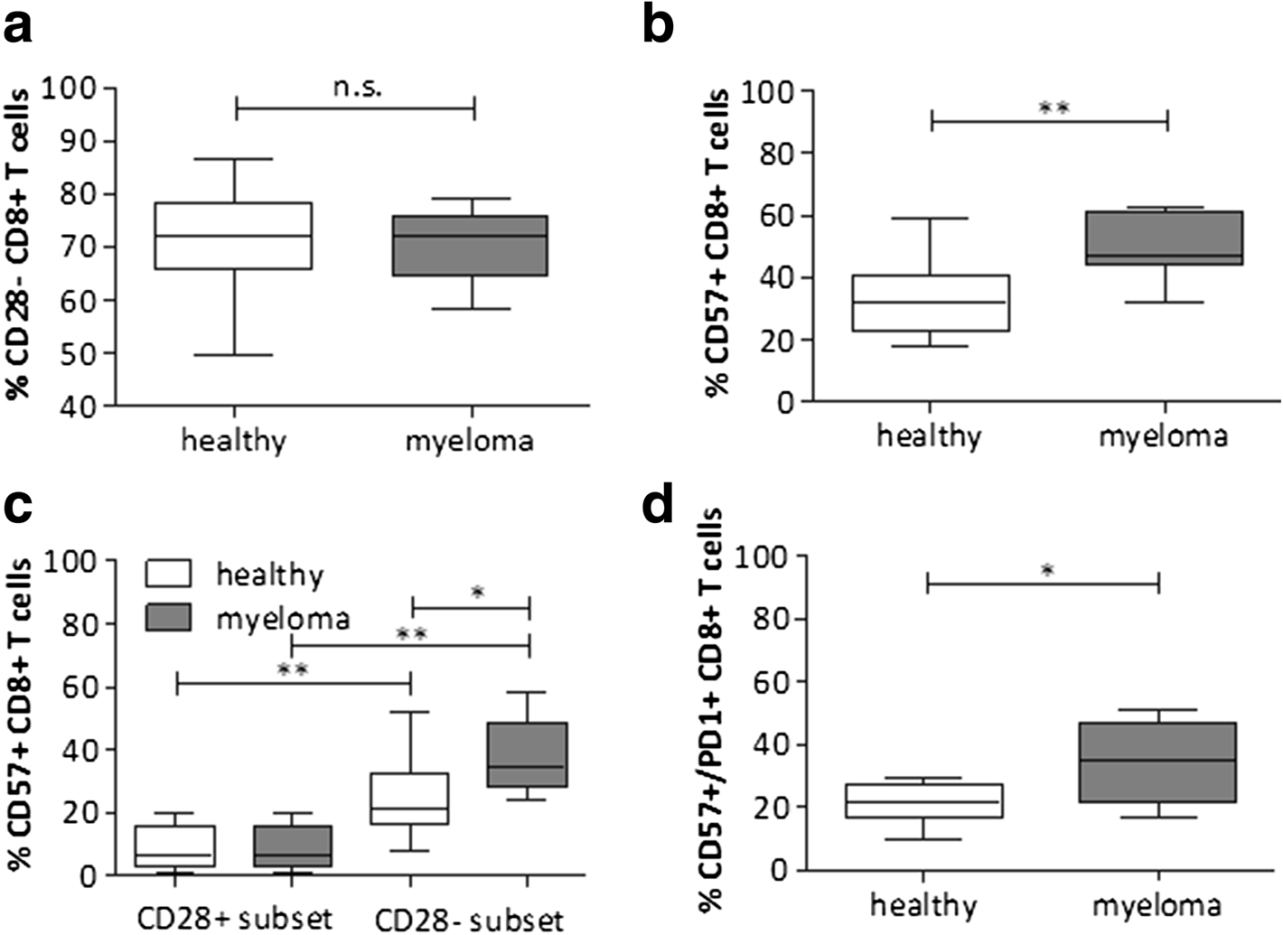
Accumulation of highly activated but senescent CD8+ T cells in the BM of myeloma patients. The expression of CD28 and CD57 was assessed on CD8+ T cells from patients with multiple myeloma and healthy, age-matched controls. Percentages of **a** CD8+CD28− T cells or **b** CD8+CD57+ T cells in healthy (*n* = 12) and myeloma samples (*n* = 12). **c** Percentages of CD8+CD57+CD28− T cells compared to CD8+CD57+CD28+ T cells (*n* = 10). **d** Concomitant expression of CD57 and PD-1 on CD8+ T cells of the BM of healthy and diseased (*n* = 6–8). Median with IQR is shown. Significance levels *p < 0.05; **p < 0.01; ***p < 0.001; *n.s.* not significant

Under persistent antigenic stimulation, CD28 expression is progressively and irreversibly downregulated, whereas the expression of CD57 increases, characterizing a state of replicative senescence [34]. Since high numbers of senescent cells might additionally blunt immunotherapies, we analyzed the extent of CD28 and CD57 expression in BM T cells [35]. In both healthy and diseased samples, most T cells lacked CD28 expression (Fig. 3a) whereas CD57 expression was significantly increased in myeloma bone marrow T cells (Fig. 3b). Specifically, the total amount of CD57+CD28− was significantly upregulated in myeloma compared to healthy BM indicating the predominance of a senescent phenotype (Fig. 3c). In addition, about 20 % of healthy and approximately 35 % of diseased CD8+ T cells co-expressed CD57 and PD-1. This enhanced co-expression in myeloma suggests a distinct population of late differentiated, senescent T cells (Fig. 3d).

### Inhibitory molecules remain expressed after therapy while CD57 is differentially expressed on CD8+ T cells in chemo-naive and treated myeloma patients

**Fig. 4:**
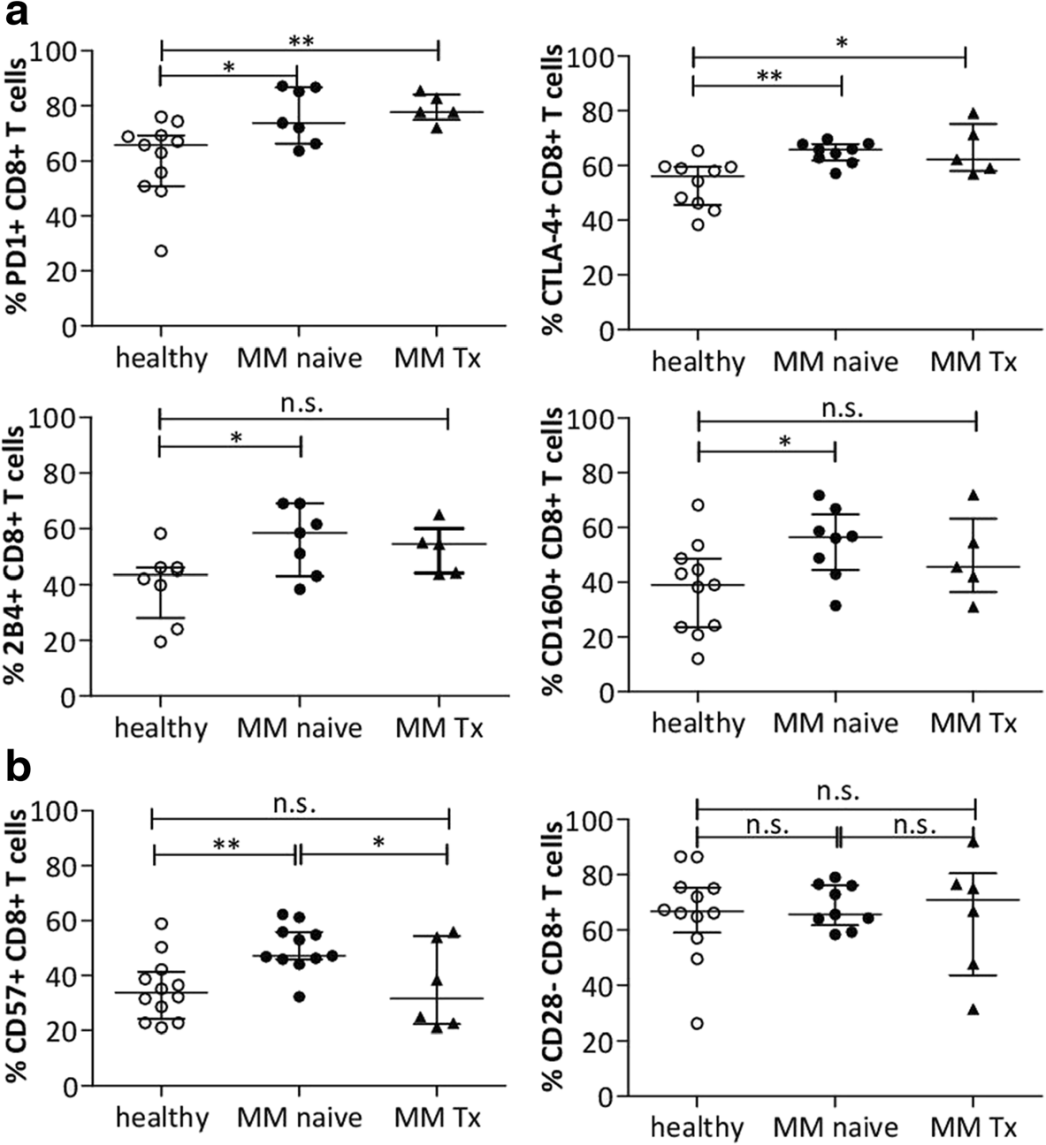
Differential expression of T cell markers in BM of newly diagnosed and refractory myeloma patients. **a** Percentage of cell surface expression of exhaustion markers PD1, CTLA-4, CD160, 2B4 was quantified on CD8+ effector T cells from patients with newly diagnosed myeloma (MM naive) and treated myeloma (MM Tx) compared to healthy donors. **b** Similar, expression of senescent markers CD57 and CD28 was investigated by flow cytometry. (Healthy *n* = 7–12; myeloma newly diagnosed *n* = 7–11; treated myeloma *n* = 5–6; median with IQR is shown). Significance levels *p < 0.05; **p < 0.01; *n.s.* not significant

So far, few investigations on the impact of established therapies on the expression of inhibitory molecules or senescence markers have been performed [15]. Therefore, we assessed surface expression of PD-1, CTLA-4, CD160, and 2B4, as well as expression of CD57 and CD28 in myeloma CD8+ T cells before and after therapy with immunomodulatory drugs and dexamethasone. PD-1 and CTLA-4 remained significantly upregulated in treated patients whereas we observed a trend for downregulation of 2B4 and CD160, although this was not significant (Fig. 4a). Notably, the analysis of treated patients pointed towards a decline of the senescent CD57+CD28− T cell population (Fig. 4b). Certainly, more data are needed to confirm these findings.

We also analyzed the percentage of CD3+ T cells which was lower in treated patients compared to chemo-naive patients. In detail, the percentages of CD4+ as well as CD8+ T cells were lower in chemo-naive patients compared to the treated ones. The CD4+/CD8+ ratio in treated myeloma patients was also lower, although results did not reach statistical significance (*p* = 0.06) (Additional file 1: Figure S1). The distribution of T cell subsets was not significantly altered except for the decrease of Temra cells in treated patients (*p* < 0.05; Additional file 2: Figure S2a). Additionally, we analyzed T cell subset distribution in untreated patient samples in correlation to the International Staging System (ISS). Although sample numbers were low, we identified changes in the T cell subsets (Tem and Temra) rather early in disease development in stage I, which were consistent also in higher stages (Additional file 2: Figure S2b). To complete our studies, we also tested inhibitory markers’ expression on CD4+ T cells from PB and BM and also CD8+ T cells from PB of treated myeloma patients. We found no significant differential expression between naive and treated CD4+ T cells except for CD57, which was also downregulated on PB CD8+ T cells (Additional file 3: Figure S3a–c).

**Fig. 5:**
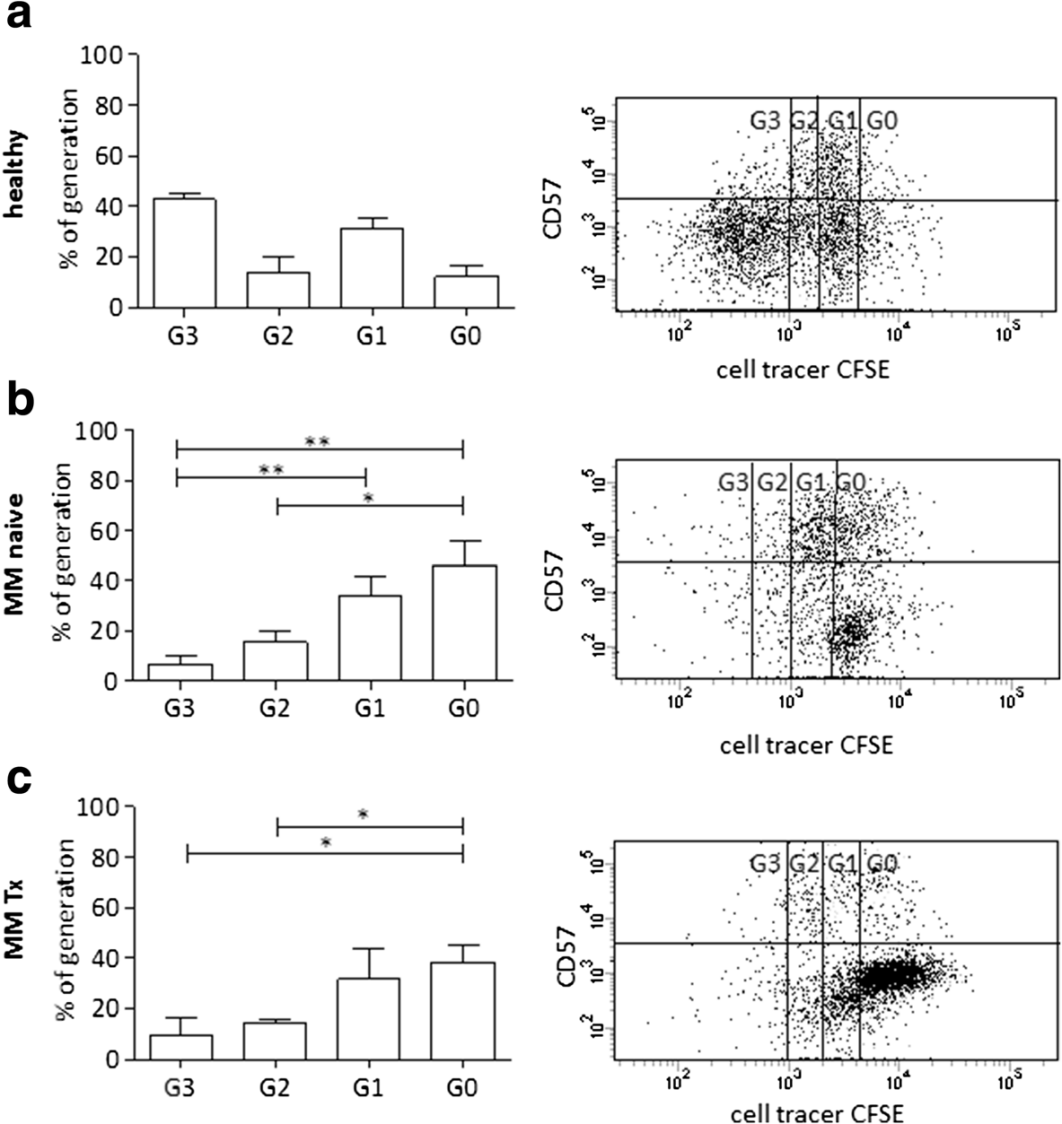
Proliferation of T cells in myeloma BM expressing CD57. Proliferation was determined by measuring dilution of CFSE using flow cytometry. Representative plots are shown for CD8+ T cells of (**a**) a healthy person, **b** a naive, and (**c**) a treated myeloma patient. The *graphs* display the results of independent experiments performed on samples from **a** four healthy persons, **b** seven chemo-naive, and **c** three treated patients (who were off IMiD-containing therapy for at least 6 months), showing the percentage of CD8+ T cells within each cell division. On the *x*-axis, the number of cell divisions according to CFSE dilution is indicated (G0 undivided; G1-3 1–3 cell divisions; significance levels *p < 0.05; **p < 0.001)

The CD8+CD57+CD28− phenotype may represent cytotoxic T cells in myeloma which are terminally differentiated, i.e., senescent. It is thought that these cells lose their ability to proliferate under in vitro stimulation [36, 37]. Therefore, we stimulated T cells from healthy donors, chemo-naive myeloma patients, and treated patients and measured proliferation and CD57 expression. As expected, healthy T cells showed a higher proliferation rate undergoing up to three cell divisions. During proliferation, they lost CD57 expression compared to chemo-naive myeloma T cells (Fig. 5a–c). However, proliferation rate, although rather low in myeloma samples, was about the same in chemo-naïve versus treated myeloma samples and CD8+ T cells retained comparable expression of CD57 (Fig. 5b, c).

Additionally, we observed enhanced overall survival in chemo-naive patients with high percentage of CD8+CD28–CD57+ T cells in the bone marrow (preliminary observations, not shown). These data go along with observations from Suen et al. [3], but certainly more patients’ data are needed before drawing definite conclusions.

## Discussion

In this study, we investigated the nature of the T cell defects in multiple myeloma in more detail and compared our findings to healthy, age-matched donor samples. Such a thorough study of BM and PB samples from myeloma patients and healthy individuals has, to our knowledge, not been performed so far. Our investigation of systemic and local changes of the expression of checkpoint inhibitor molecules PD-1, CTLA-4, CD160, and 2B4 revealed significant upregulation of all tested markers especially in the bone marrow, suggesting that the immunosuppressive tumor microenvironment fuels immune escape in multiple myeloma.

Expression and ligation of immune-checkpoint molecules downregulate T cell responses and thereby maintain self-tolerance. The use of monoclonal antibodies (i.e., anti-PD-1) to disrupt receptor-ligand interactions has already shown remarkable results in the therapy of several solid tumors [38–40] and is currently a fast progressing field also in hematological cancers including myeloma. A major obstacle is the profoundly suppressive tumor microenvironment which limits actions of immune cells against cancer [41]. Coupled with the finding that myeloma cells express PD-L1 which can even be further upregulated by stroma cell contact, the PD-1 pathway has been shown to contribute to the aggressiveness of this disease in a subset of patients [14]. Although in vitro results strongly support PD-1 inhibition as novel, effective therapy [16], these drugs failed to induce major responses in myeloma in vivo so far, as compared to other B cell malignancies [13]. Here, the tumor microenvironment emerges as important additional target and ongoing studies which combine PD-1 inhibitors with immunomodulatory agents (i.e. lenalidomide and pomalidomide) appear to be more successful. Lenalidomide has been shown to induce immuno-activating changes in the myeloma microenvironment [15, 42] and to downregulate PD-L1 expression [14]. However, since PD-L1 expression per se varies strongly in myeloma [43], other checkpoint inhibitors might become equally important targets. Studies in the 5T33 murine myeloma model already showed the efficacy of lenalidomide in combination with blockade of other immune checkpoints (e.g. CTLA-4, LAG-3, TIM-3, and combinations thereof [44]). Our analyses show that CTLA-4, 2B4, and CD160 expression remains high even after IMiD-containing therapies and thus could constitute additional novel targets. CTLA-4 competes with the immunostimulatory receptor CD28 for the binding of CD80/CD86 proteins. Tumor engagement of the CTLA-4 pathway may therefore dampen the immune response in the microenvironment resulting in an inappropriate T cell costimulation [45]. In fact, ipilimumab, an antagonistic antibody of CTLA-4, was the first immune-checkpoint inhibitor studied in lymphoid malignancies with promising results [46]. However, investigations in multiple myeloma are still pending. CD160, one of the five ligands of herpes virus entrance mediator (HVEM) has the potential to shift immune response towards exhaustion, and its expression has been shown to be independent of PD-1 [26]. Moreover, CD160 blockade has not been investigated in myeloma so far. CD28 downregulation and 2B4 upregulation in the presence of CTLA-4 has been shown in a virus-specific T cell model [47]. We here see a similar situation in myeloma bone marrow, i.e., a high proportion of CD8+ T cells which lost CD28 expression and concomitantly gained CTLA-4 expression and upregulated 2B4. These regulations could be the result of chronic stimulation leading to enhanced T cell exhaustion. The CMV status of the patients might play a role here, however, recent papers showed defects in T cell function irrespective of CMV serostatus [48, 49].

Additionally, to our checkpoint molecule analysis, we detected an altered T cell subset distribution at the tumor site, leading to a significant increase of the effector T cell population at the expense of the memory T cell population. These CD8+ BM T cells would generally favor anti-cancer immune responses. However, these cells also displayed upregulation of checkpoint molecules, reduced cytokine production, reduced ability to degranulate in response to T cell stimulation, and reduced proliferative capacity. Thus, without further activations, these T cells are most likely ineffective in tumor immune surveillance. The loss of CD28 expression is a sign of T cell aging in healthy individuals [30], and we found it further enhanced in myeloma patients. In addition, we observed that myeloma CD8+CD28− T cells concomitantly expressed CD57, pointing to a high accumulation of a late differentiated, senescent T cell population in the BM. CD8+CD28–CD57+ T cells from peripheral blood have been shown to play an active pro-tumor role via suppressing proliferation of responder T cells [34], and removal of these cells in vitro restored T cell proliferative capacity [50, 51]. Moreover, CD8+CD28–CD57+ T lymphocyte clones in the PB of myeloma patients have been associated with progressive and advanced stage disease [52]. In our analyses, we found that the percentage of CD57+ T cells in the bone marrow of treated patients was reduced suggesting a window of opportunity for effective immune modulatory treatments. However, sample size needs to be extended to be able to draw solid conclusions about the potential role of CD8+CD28–CD57+ T cells in myeloma. Clearly, due to the severe impairment of T cell function, reactivation of T cells has to be built on several columns. An additional promising approach demonstrated that exogenous IL-7 added to T cells co-cultured with tumor cells inhibited loss of CD28, a feature of replicative senescence, and allowed normal proliferative capacity and IL-2 production [53]. IL-7 can also induce telomerase activity [54]. Interestingly, an ongoing phase I clinical trial using the non-glycosylated form of human IL-7 shows promise, in that it seems to cause the expansion of naive and memory CD4 and CD8 T cell populations (NTC00062049). This may be important for myeloma patients as especially memory T cells are reduced at the tumor site. Together with IL-15, which is a critical factor for development, proliferation and activation of natural killer cells and CD8+ memory T cells, it may effectively contribute to fight the tumor. In preclinical studies, this cytokine exhibited potent antitumor activities against established tumors in animal models, showing that CD8+ T cells play a pivotal role in the anti-myeloma effect of IL-15 agonists [55, 56].

## Conclusions

In this study, we show that the percentages of CD8+ effector T cells especially at the tumor site, i.e., the bone marrow, of myeloma patients are increased but cells are functionally severely impaired and display several features of exhaustion and senescence. Whereas expression of checkpoint inhibitory molecules is retained during the course of treatment, senescent T cell-marker CD57 appears to be downregulated. These data clearly show that several avenues for the reactivation of the immune response in multiple myeloma will finally be needed to develop novel successful therapies.

## Additional files

Additional file 1: Figure S1. T cell distribution in BM aspirates from myeloma patients and healthy persons. (TIFF 44 kb)
Additional file 2: Figure S2a. T cell subset distribution in BM aspirates of naive and refractory myeloma patients compared to healthy individuals. **Figure S2b.** T cell subset distribution in BM aspirates of naive myeloma patients **(subdivided according to ISS staging)** compared to healthy individuals. (TIFF 100 kb)
Additional file 3: Figure S3. Differential expression of exhaustion and senescent markers in T cells in myeloma BM and PB of newly diagnosed and treated patients. Expression on CD4+ T cells in the bone marrow (**Figure S3a**), CD4+ T cells in PB (**Figure S3b**), and CD8+ T cells in PB (**Figure S3c**). (ZIP 192 kb)

## Abbreviations

BM: Bone marrow
BMMC: Bone marrow mononuclear cells
CTLA-4: Cytotoxic T lymphocyte-associated protein 4
PB: Peripheral blood
PBMC: Peripheral blood mononuclear cells
PD-1: Programmed cell death protein 1
