# Supplementary figures and images for: T cells in multiple myeloma display features of exhaustion and senescence at the tumor site
Source: J Hematol Oncol. 2016 Nov 3;9:116. doi: 10.1186/s13045-016-0345-3 (PMC5093947; doi:10.1186/s13045-016-0345-3)

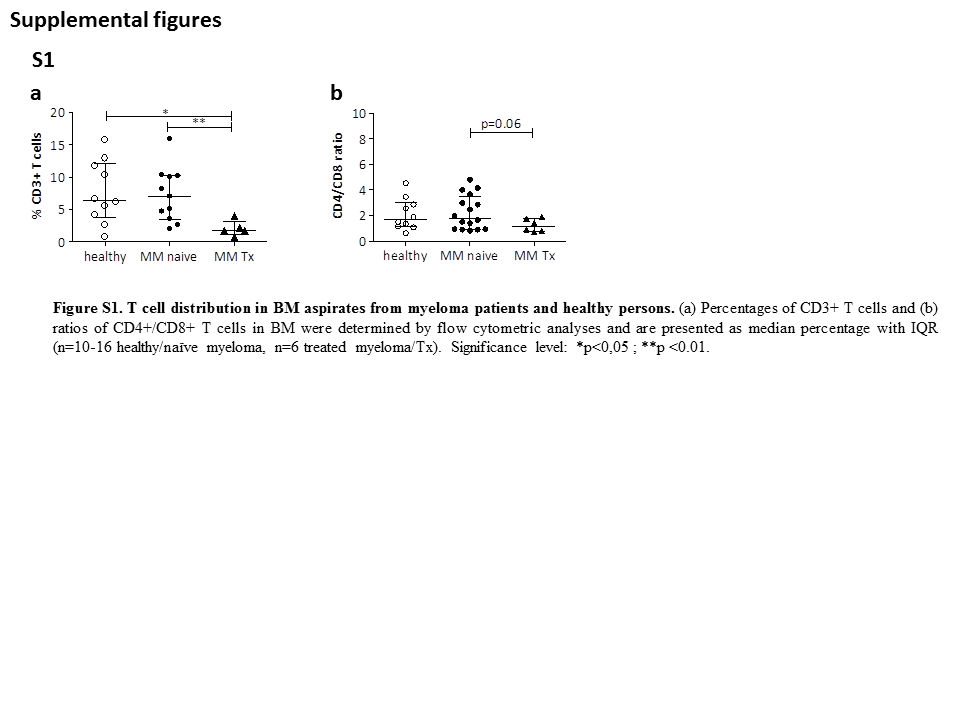

Supplement: Additional file 1: Figure S1. — T cell distribution in BM aspirates from myeloma patients and healthy persons. (TIFF 44 kb) [file 13045_2016_345_MOESM1_ESM.tif]

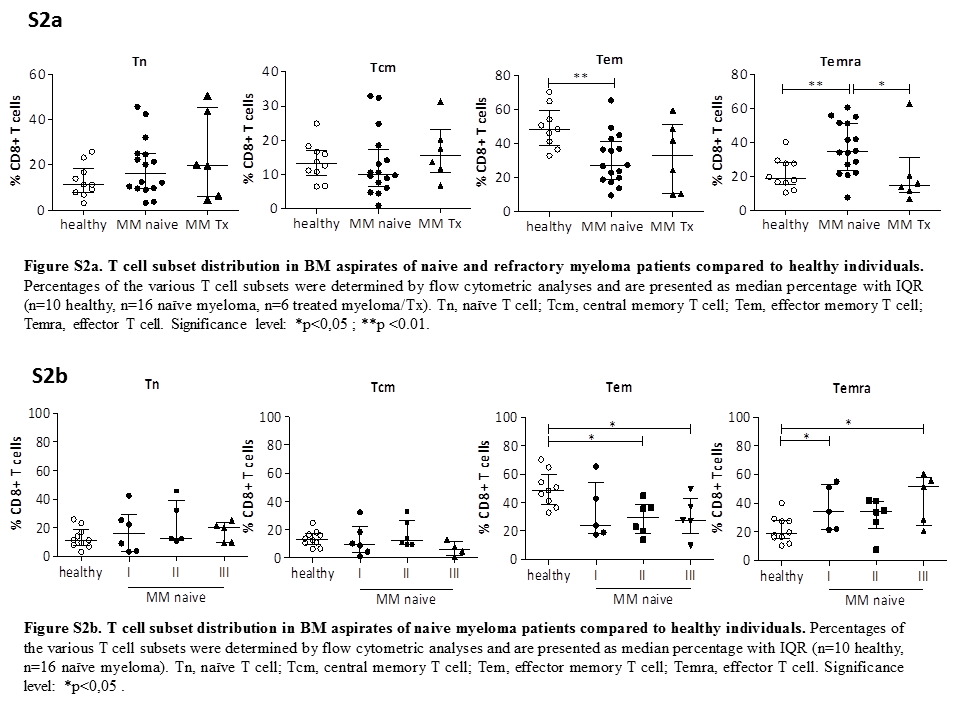

Supplement: Additional file 2: Figure S2a. — T cell subset distribution in BM aspirates of naive and refractory myeloma patients compared to healthy individuals. Figure S2b. T cell subset distribution in BM aspirates of naive myeloma patients (subdivided according to ISS staging) compared to healthy individuals. (TIFF 100 kb) [file 13045_2016_345_MOESM2_ESM.tif]

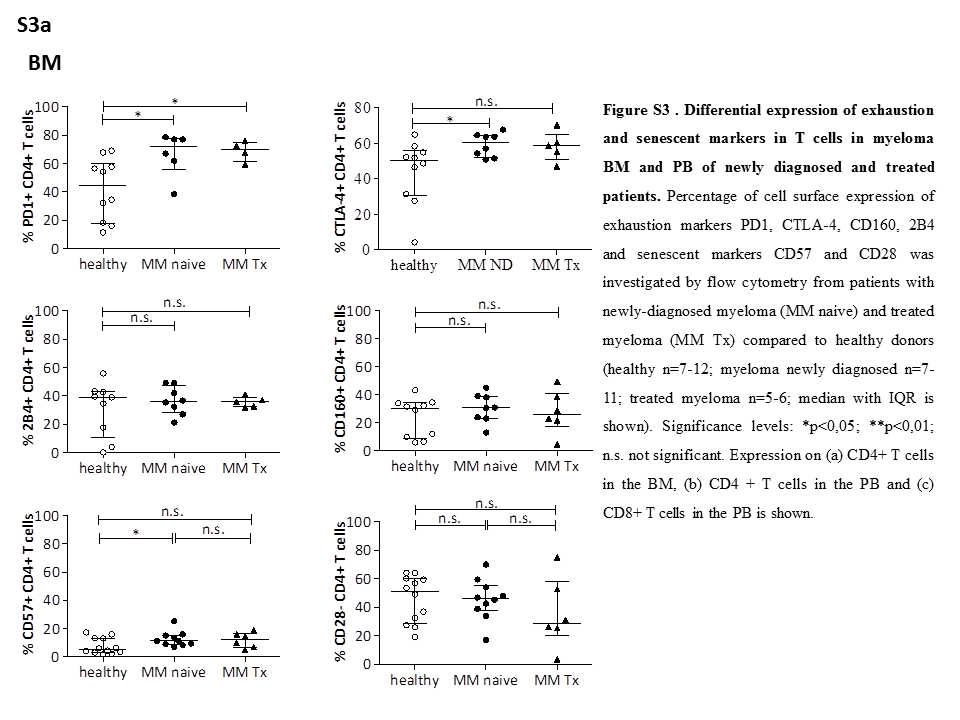

Supplement: Additional file 3: Figure S3. — Differential expression of exhaustion and senescent markers in T cells in myeloma BM and PB of newly diagnosed and treated patients. Expression on CD4+ T cells in the bone marrow (Figure S3a), CD4+ T cells in PB (Figure S3b), and CD8+ T cells in PB (Figure S3c). (ZIP 192 kb) [file 13045_2016_345_MOESM3_ESM.zip › supplemental figure 3a.tif]

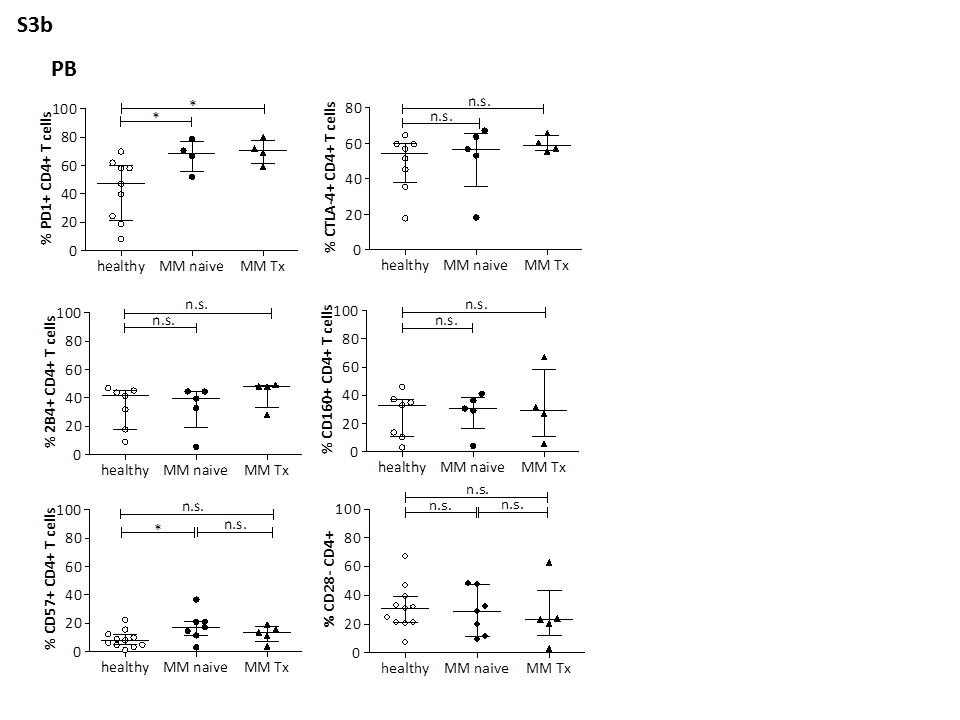

Supplement: Additional file 3: Figure S3. — Differential expression of exhaustion and senescent markers in T cells in myeloma BM and PB of newly diagnosed and treated patients. Expression on CD4+ T cells in the bone marrow (Figure S3a), CD4+ T cells in PB (Figure S3b), and CD8+ T cells in PB (Figure S3c). (ZIP 192 kb) [file 13045_2016_345_MOESM3_ESM.zip › supplemental figure 3b.tif]

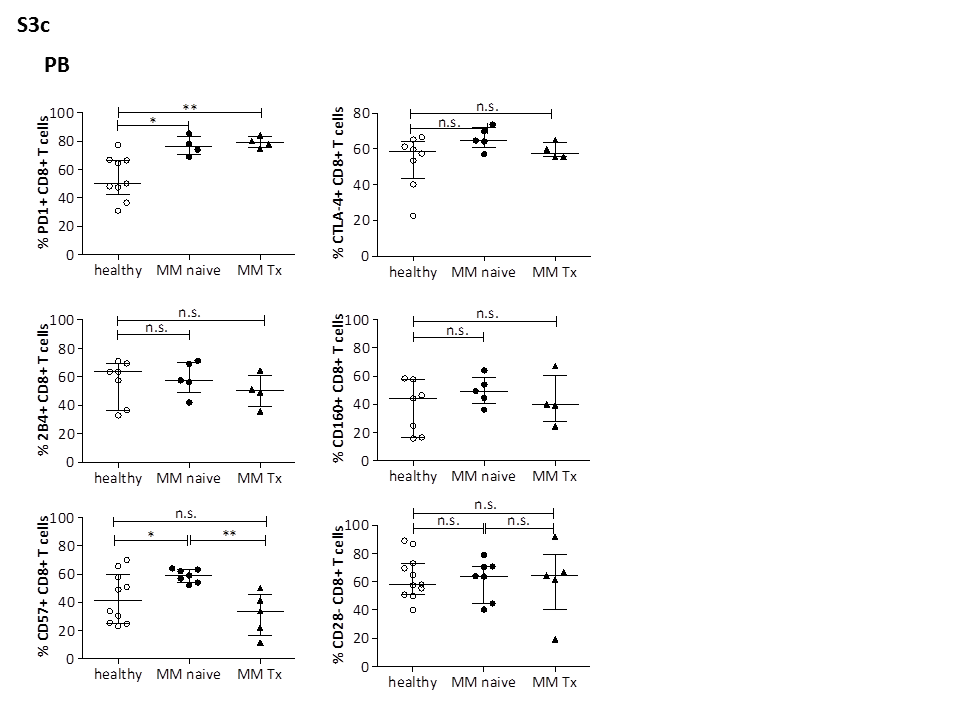

Supplement: Additional file 3: Figure S3. — Differential expression of exhaustion and senescent markers in T cells in myeloma BM and PB of newly diagnosed and treated patients. Expression on CD4+ T cells in the bone marrow (Figure S3a), CD4+ T cells in PB (Figure S3b), and CD8+ T cells in PB (Figure S3c). (ZIP 192 kb) [file 13045_2016_345_MOESM3_ESM.zip › supplemental figure 3c.tif]
